# Data from an incentivized laboratory experiment on strategic medical choices

**DOI:** 10.1016/j.dib.2021.106926

**Published:** 2021-03-03

**Authors:** Ge Ge, Geir Godager

**Affiliations:** a Institute of Health and Society, Department of Health Management and Health Economics, University of Oslo, Norway; b Health Services Research Unit, Akershus University Hospital, Norway

**Keywords:** Incentivized laboratory experiment, Oligopoly, Competition, Behavioral game theory, Quantal response equilibrium, Physician behavior

## Abstract

This paper presents data of medical choices determining physicians’ profit and patients’ health benefit under three levels of market competition: monopoly, duopoly, and quadropoly. The data was collected from 136 German university students in an incentivized laboratory experiment. The designed experimental parameters and the formula for computing the payoff matrices of the games are described in this paper as well. This data was analyzed by Ge and Godager [5] who employed quantal response equilibrium choice  models to investigate the relationship between market competition and determinism in behavior under a quantal response equilibrium paradigm. This data contributes to future investigation on alternative game theoretic equilibrium concepts and the development of empirical methods for studying strategic choice behavior.

## Specifications Table

**Table:** 

Subject	Economics and Econometrics
Specific subject area	Behavioral game theory
Type of data	Table
How data were acquired	Incentivized laboratory experiment programmed and implemented using zTree
Data format	Raw, Partially analyzed
Parameters for data collection	Strategic choices in Monopoly, Duopoly and Quadropoly market settings
Description of data collection	Data was collected in 5 experimental sessions at Cologne Laboratory for Experimental Research of the University of Cologne
Data source location	University of Cologne, Cologne, Germany
Data accessibility	With the article
Related research article	Ge, Ge, and Geir Godager. “Predicting strategic medical choices: An application of a quantal response equilibrium choice model.” *Journal of Choice Modelling* (2021): 100282.

## Value of the Data

• The data enables the analysis of strategic medical choices that determine profit and health benefit for real patients.
• This data can be beneficial for researchers interested in incentivized choice experiments and behavioral game theory.
• The data can be useful for exploring several alternative game theoretic equilibrium concepts, the development of new empirical methods and teaching.

## 1 Data Description

The data includes 136 subjects’ medical choice decisions under three market conditions. The three market conditions, namely *monopoly, duopoly*, and *quadropoly* have various levels of competition where 1, 2, and 4 players make strategic choices simultaneously, respectively. Decisions are made for 8 “patients” in each market condition, hence there are 24 games in total in the experiment. In each game, the subjects choose among 11 medical treatments that determine their own profit and patients’ health benefit. In duopoly and quadropoly, the subjects are randomly matched, and the joint decisions by the matched group determine their payoffs. The random match is dissolved after completion of all the decision tasks in each market setting.

The complete choice data of the 136 subjects is presented in the Excel file *GeGodager_2020_rawdata.xlsx*. Table 1 below summarizes the frequencies of each strategy being chosen in each game.

**Table 1:** Observed frequencies of strategy choice in the 24 patient games by the 136 subjects.

		Pure strategy										
Market	Patient game	0	1	2	3	4	5	6	7	8	9	10
**Monopoly**	1	24	12	4	18	14	27	16	15	2	2	2
	2	21	9	9	13	11	18	24	14	10	1	6
	3	23	6	10	9	11	14	10	22	14	6	11
	4	23	5	7	13	11	19	9	29	11	3	6
	5	24	7	7	19	18	15	14	21	5	4	2
	6	23	4	8	21	14	17	11	15	13	4	6
	7	21	6	7	14	7	14	11	13	24	7	12
	8	21	4	14	12	7	16	14	8	20	7	13

**Duopoly**	1	0	0	1	3	3	12	24	41	26	22	4
	2	0	0	0	2	4	7	12	27	36	26	22
	3	0	0	0	3	4	3	9	18	37	34	28
	4	1	0	3	1	0	4	6	21	30	29	41
	5	1	0	0	1	4	18	22	48	32	7	3
	6	1	0	0	1	4	9	14	33	42	19	13
	7	1	1	0	1	1	1	3	15	18	28	67
	8	0	1	0	0	1	2	7	14	25	28	58

**Quadropoly**	1	0	0	0	2	2	4	14	31	48	30	5
	2	0	0	0	2	0	3	10	11	42	31	37
	3	1	0	0	0	0	0	3	12	26	41	53
	4	1	0	1	0	0	0	4	9	24	39	58
	5	0	0	0	0	0	10	9	37	58	17	5
	6	0	0	0	1	1	1	8	15	40	43	27
	7	0	0	0	1	0	1	3	2	16	22	91
	8	0	0	0	0	1	1	4	5	14	27	84

## 2 Experimental Design, Materials and Methods

An incentivized laboratory experiment was conducted at University of Cologne. The experiment has a medical framing and the participants are instructed to play the role of a physician and choose medical treatment for eight “patients” in each of three different market settings; monopoly, duopoly and quadropoly. The treatment choices of participants determine their own profit and patients’ health benefit. The profit and patient benefit accrued in the laboratory are converted into monetary transfers to the participants and a charity dedicated to providing surgeries for ophthalmic patients, respectively. This element of our protocol, which is identical to Hennig-Schmidt et al. [4], motivates participants’ patient-regarding behavior in the laboratory. The sessions lasted about 90 minutes on average, and participants earned on average about 14 Euros and provided about 8 Euros patient benefits. In total, 1 102 Euros were transferred to the Christoffel Blindenmission. The experiment was programmed in zTree [1] and the subjects were recruited through ORSEE [2].

### 2.1 Construction of payoff matrices

Physicians receive profit by treating patients and patients gain health benefit. In each market, there is a fixed demand of 100 patients of the same type. In monopoly, the physician serves the whole market, while in duopoly and quadropoly, the demand is assumed to respond positively to the patient benefit from the treatment. In other words, in the competitive markets, the physician who provides larger health benefit is more likely to attract more patients, and his market share is therefore positively related to the health benefit he provides and negatively related to the health benefit provided by his opponent(s). On the other hand, providing larger health benefits increases costs and hence reduces physician’s profit margin. In each game, the payoffs are constructed based on the specified demand function and the experimental parameters designed to characterize a “patient type”. In total, there are eight patient types and 24 games. In the following, we describe in details the experimental parameters, the specifications of the market demand function and the computation of the payoff elements. Interested readers can reproduce the payoff matrices using Stata code *Data_in_Brief.do* presented in the supplementary materials.

#### 2.1.1 Experimental parameters

We use three parameters, F, ϕ, and δ, to characterise different patient types. The capitation payment parameter, F, denotes the payment to the physician for each patient he treats, and takes the value of either 10 or 15. The cost parameter, ϕ, specifies the cost of the treatment, and is either 0.075 or 0.1. The patient benefit parameter, δ, denotes the benefit the patient receives, and is either 0.5 or 1. The 2×2×2 combinations of parameter levels make up a total of eight unique configurations characterizing eight patient types for each market in the experiment.

Consider a choice set C of 11 treatment strategies (C={0,1,2…10}). For any strategy j from the choice set C (j∈C), we denote per-patient profit as $\pi_{j},$ and per-patient benefit as $b_{j},$ and they are given by:

(1a) $$\pi_{j}=F-\phi j^{2},$$

(1b) $$b_{j}=\delta j.$$

Table 2 below describes variation in per-patient profit and per-patient benefit over the eight patient types.

**Table 2:** Per-patient profit and per-patient benefit over patient types, j=0,1,2…10.

Patient Type	$\pi_{j}$	$b_{j}$
1	$10-0.1j^{2}$	j
2	$10-0.075j^{2}$	j
3	$15-0.1j^{2}$	0.5j
4	$15-0.1j^{2}$	j
5	$10-0.1j^{2}$	0.5j
6	$10-0.075j^{2}$	0.5j
7	$15-0.075j^{2}$	j
8	$15-0.075j^{2}$	0.5j

#### 2.1.2 Market demand function

A physician’s demand is determined by the treatment choices of all competing physicians through a logistic demand system:

(2a) $$D_{j}=100\;\text{Monopoly}$$

(2b) $$D_{j|x}=\frac{100\exp\left(b_{j}\right)}{\exp\left(b_{j}\right)+\exp\left(b_{x}\right)}\;\text{Duopoly}$$

(2c) $$D_{j|\mathrm{xyz}}=\frac{100\exp\left(b_{j}\right)}{\exp\left(b_{j}\right)+\exp\left(b_{x}\right)+\exp\left(b_{y}\right)+\exp\left(b_{z}\right)}\;\text{Quadropoly}$$

where j,x,y,z∈C. In monopoly, a physician serves the whole market, the demand of choosing j, denoted as $D_{j},$ is therefore fixed to 100 patients. In duopoly, a physician’s demand of choosing j given the opponent’s choice is x, denoted as $D_{j|x},$ is a function of his chosen per-patient benefit $b_{j}$ and the per-patient benefit $b_{x}$ chosen by the opponent. Similarly, in quadropoly, a physician’s demand of choosing j given the combination of the opponents’ choices is xyz, $D_{j|xyz},$ depends on the per-patient benefit chosen by all the four physicians in the game, $b_{j},$ $b_{x},$ $b_{y}$ and $b_{z}$. The design of this demand response reflects a potential competitive scenario among health care providers in the market.

#### 2.1.3 Payoff elements

Subjects receives a vector of payoff comprising two elements: total profit, Π, and total patient benefit, B. The total profit and health benefit of choosing strategy j, $\Pi_{j}$ and $B_{j},$ depend on per-patient-profit, per-patient-benefit and the demand. We assume that subjects value both own profit and benefits to patients. Further, a healthy patient population in the market is assumed to be a shared good and hence a physician’s valuation of patient benefit is independent of the care provider [3]. In other words, B here is the total benefit of all the patients in the market. We let $\Pi_{j}$ and $B_{j}$ denote the payoff elements from alternative j in monopoly, $\Pi_{j|x}$ and $B_{j|x}$ denote the payoff elements from choosing j given the opponent’s choice is x in duopoly, and similarly $\Pi_{j|xyz}$ and $B_{j|xyz}$ denote the payoff elements from choosing j given the combination of the opponents’ choices is xyz in quadropoly. A physician’s payoffs from choosing j given the opponent(s)’ choice(s) can be expressed as:

(3a) $$\Pi_{j}=D_{j}\pi_{j}\;\text{Monopoly}$$

(3b) $$B_{j}=D_{j}b_{j}\;\text{Monopoly}$$

(3c) $$\Pi_{j|x}=D_{j|x}\pi_{j}\;\text{Duopoly}$$

(3d) $$B_{j|x}=D_{j|x}b_{j}+D_{x|j}b_{x}\;\text{Duopoly}$$

(3e) $$\Pi_{j|xyz}=D_{j|xyz}\pi_{j}\;\text{Quadropoly}$$

(3f) $$B_{j|xyz}=D_{j|xyz}b_{j}+D_{x|jyz}b_{x}+D_{y|jxz}b_{y}+D_{z|jxy}b_{z}\text{Quadropoly}$$

where j,x,y,z∈C.

We now illustrate with examples how to calculate physicians’ payoff (Π and B) in each market.

• Example 1: Monopoly, Patient Type 3, j=7.
  We see from Table 2, when the physician chooses j=7, he receives $15-0.1\times 7^{2}=10.1$ Taler of profit for each patient and the per-patient benefit is 0.5×7=3.5 Taler. Under monopoly, a physician serves the whole market with 100 patients. Therefore, the physician’s payoff contains 10.1×100=1010 Taler profit and 3.5×100=350 Taler patient benefit.
• Example 2: Duopoly, Patient Type 3, j=7, x=5.
  Under duopoly, the physician’s demand is determined by the per-patient benefits chosen by him and his opponent. Hence, the physician’s demand is $\frac{100\exp(0.5\times 7)}{\exp(0.5\times 7)+\exp(0.5\times 5)}$ and his opponent’s demand is $\frac{100\exp(0.5\times 5)}{\exp(0.5\times 7)+\exp(0.5\times 5)}$. The total patient benefit included in the physician’s payoff is therefore: $3.5\times \frac{100\exp(3.5)}{\exp(3.5)+\exp(2.5)}+2.5\times \frac{100\exp(2.5)}{\exp(3.5)+\exp(2.5)}=323$ Taler. The player’s profit is $10.1\times \frac{100\exp(3.5)}{\exp(3.5)+\exp(2.5)}=737$ Taler.
• Example 3: Quadropoly, Patient Type 3, j=7, x=5,y=5,z=5.
  Under quadropoly, the physician’s demand is determined by the per-patient benefits chosen by him and his three opponents. The physician’s demand is therefore $\frac{100\exp(3.5)}{\exp(3.5)+\exp(2.5)+\exp(2.5)+\exp(2.5)}$. The total patient benefit in the physician’s payoff vector is $3.5\times \frac{100\exp(3.5)}{\exp(3.5)+\exp(2.5)+\exp(2.5)+\exp(2.5)}+3\times 2.5\times \frac{100\exp(2.5)}{\exp(3.5)+\exp(2.5)+\exp(2.5)+\exp(2.5)}=298$ Taler. The total profit is $10.1\times \frac{100\exp(3.5)}{\exp(3.5)+\exp(2.5)+\exp(2.5)+\exp(2.5)}=485$ Taler.

### 2.2 Experimental procedure

Upon arrival, participants were randomly assigned to cubicles. The instructions (available in the PDF file *Instructions.pdf*) informed participants of the structure of the experiment and the payoffs. To facilitate non-cooperative decision-making, the employment of random matching and re-matching of participants was described clearly to them. The participants had available a “calculator” in the zTree program to see how a combination of treatment choices determine payoffs. In other words, the participants could inspect each cell of the payoff matrices. The sequence of the three markets to be played by the participants varied in order to mitigate the “order effects”.^1^ Participants were given adequate time to read the instructions and ask clarifying questions in private. For each market setting, participants answered control questions to make sure they understood how (joint) choices affect their profit and the patient benefit.

At the end of the experiment, one randomly drawn outcome from each market determined the participant’s payment and the patient benefits to be transferred to the charity. To ensure participants’ trust in the transfer of patient benefits, we applied a procedure similar to Hennig-Schmidt et al. [4]. One participant was chosen at random to be a *monitor*. After the experiment, the monitor verified that a money order, equivalent to the total patient benefit provided by all participants, was issued by the Finance Department of the University of Cologne. The money order was payable to the *Christoffel Blindenmission*, which supports ophthalmologists performing cataract surgeries in a hospital in Masvingo, Zimbabwe. After sealing the money order in an envelope, the monitor and an experiment assistant walked together to the nearest mailbox and deposited the envelope. The monitor was paid an additional 5 Euros.

## Ethics Statement

The ethical review and approval of experimental procedure was given by Norwegian Social Science Data Services (reference number 43709). Informed consent was obtained orally from all subjects.

## Declaration of Competing Interest

The authors declare that they have no known competing financial interests or personal relationships which have, or could be perceived to have, influenced the work reported in this article.

## Appendix A Supplementary materials

Supplementary Data S1 Stata commands. Supplementary Raw Research Data. This is open data under the CC BY license http://creativecommons.org/licenses/by/4.0/ Supplementary Data S1

Supplementary Data S2 Raw data. Supplementary Raw Research Data. This is open data under the CC BY license http://creativecommons.org/licenses/by/4.0/ Supplementary Data S2

Supplementary Data S3 Instructions for participants of the experiment. Supplementary Raw Research Data. This is open data under the CC BY license http://creativecommons.org/licenses/by/4.0/ Supplementary Data S3

## Footnotes

1 Among all 136 participants, 56 played in the order of monopoly, duopoly, then quadropoly; 28 played in the order of monopoly, quadropoly, then duopoly; 24 played in the order of duopoly, quadropoly, and monopoly; and 28 played in the order of quadropoly, duopoly, then monopoly.
