# Supplementary material for: Data from an incentivized laboratory experiment on strategic medical choices
Source: Data Brief. 2021 Mar 3;35:106926. doi: 10.1016/j.dib.2021.106926 (PMC7988277; doi:10.1016/j.dib.2021.106926)
Supplement: Supplementary Data S3 — Instructions for participants of the experiment. Supplementary Raw Research Data. This is open data under the CC BY license http://creativecommons.org/licenses/by/4.0/ [file mmc3.pdf]

Instructions for participants of the experiment

You are taking part in an economic decision-making experiment. Please carefully read the instructions. It is very important that you do not speak with other participants for the duration of the experiment. If you break these rules, you could be excluded from the experiment and not receive any payment. If you do not understand something, please take another look at the instructions. If you still have questions, please raise your hand. We will come to you at your cubicle and answer your questions in private.

You can earn money in the course of the experiment. The amount of your earnings depends on your decisions and decisions made by other participants. At no time will you be told the names of the other participants. They will also not at any time be informed about your identity.

For showing up you will receive a fee of EUR 2.50.

All monetary amounts in this experiment are expressed in Taler, whereby the following applies: Taler 100 = EUR 1.

At the end of the experiment, the amount of money you earned will be paid to you in cash. Your decisions are made on the computer screen present in your cubicle. All data and answers will be evaluated anonymously. You were asked to draw your own personal cubicle number in order to maintain anonymity.

The experiment will last around 60 minutes and consists of three parts. Before each of the three parts you will receive detailed instructions and be asked to answer control questions pertaining to these instructions. Please note: Neither your decisions in the first part nor in the second part of the experiment have an influence on the other parts of the experiment.

We will ask you to answer a few questions at the end of the experiment. You will receive an additional payment for answering this questionnaire.

First part of the Experiment. In the first part of experiment, you will take on the role of a physician and make decisions about the treatment of various patients. In total, you will determine the quality of care that you would like provide for eight different types of patients. For each of these patients you can choose quality of 0, 1, 2, 3, 4, 5, 6, 7, 8, 9 or 10.

The demand for medical care by the various patient types is determined only after you have made your decisions about the quality of care for all eight types.

[Duopoly: You are randomly matched with another participant. This participant also decides

in the role of a physician. Also this physician determines the quality for the same eight types of patients. The matching with this participants remains throughout the entire second part of the experiment. You and the other physician choose the quality simultaneously and independently from each other.]

[Quadropoly: You are randomly matched with three other participants. These participants also decide in the role of a physicians. Also these physicians determine the quality for the same eight types of patients. The matching with these participants remains throughout the entire third part of the experiment. You and the other physicians choose the quality simultaneously and independently from each other.]

In total, 100 patients of each type demand medical care. It will only be determined after you have made your decisions about the quality of care for all eight types how many of the 100 patients of each type who seek treatment from you.

[Duopoly: Only after you and the other physician, you are matched with, decided upon the quality of medical treatment for the eight patients, it is determined how many of the 100 patients seek treatment from you and the other physician.]

[Quadropoly: Only after you and the others physicians, you are matched with, decided upon the quality of medical treatment for the eight patients, it is determined how many of the 100 patients seek treatment from you and the other physicians.]

Earnings. For each patient who seeks medical care from you, you receive a lump sum that is independent of the quality of care you have selected. You incur costs with your selection of the quality of care. These costs depend on the quality level you choose and can vary between the different patient types. Your earnings for each patient type are as follows:

$$\text{Earnings} = (\text{Lump sum} - \text{Costs}) \times \text{Number of patients who seek medical care from you}$$

(when read: your earnings are equal to the difference between the lump sum and the costs that arise from the quality of care you have chosen, multiplied by the number of patients who seek treatment from you.)

With the quality of care you choose, you determine not only your own earnings, but also the utility enjoyed by the patient. The amount of the lump sum, your costs, your earnings, and the patient's utility will be displayed on your screen (as illustrated below) for each patient type. Before you choose the quality of care for each patient type, you have the opportunity to click on the "calculator" button and thereby calculate patients' potential demand for treatment (as illustrated below). You can enter the quality you would like

[Duopoly: 2nd example screen]

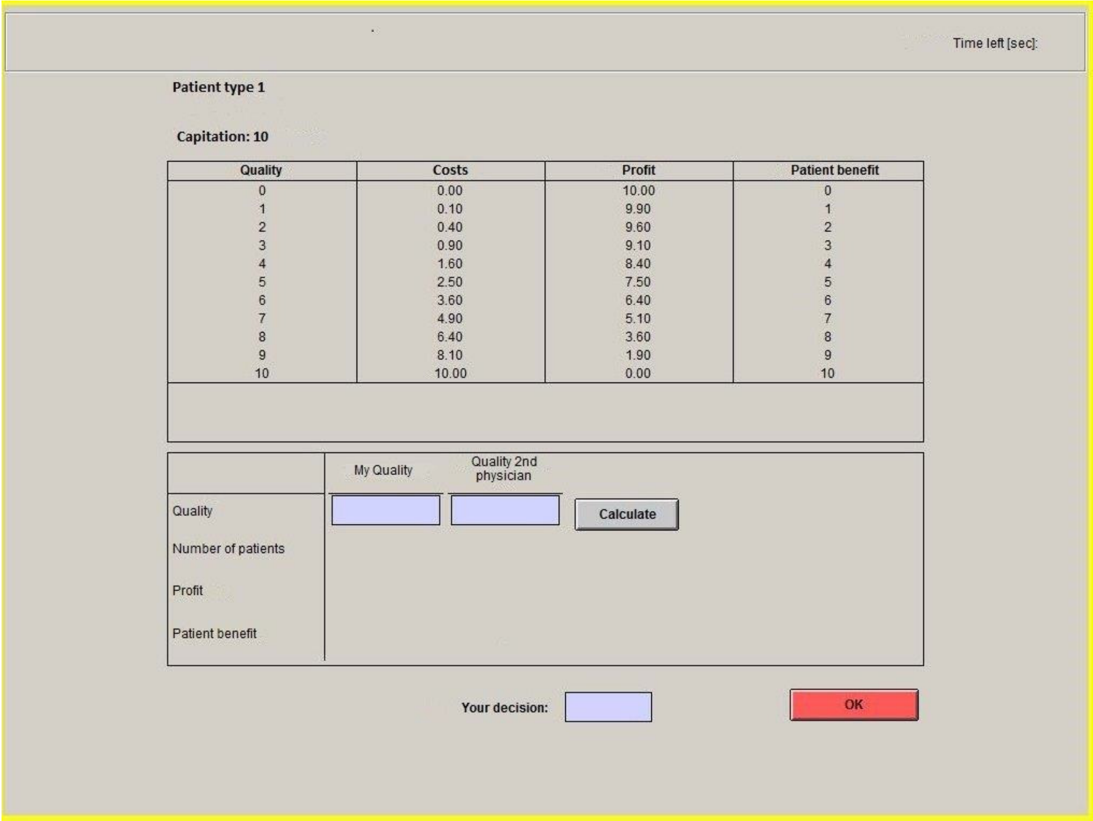

to provide as many times as you want. Clicking on the "calculate" button provides you with information about the number of patients who would seek care given the quality level you entered. In addition, you receive information about the resulting earnings and patient utility. You define the quality of care that you wish to provide by entering that quality in the field "your decision" and confirming this entry with "OK."

Payment. After the conclusion of the experiment, one of the 8 decisions will be randomly chosen to function as the relevant round for determining your payment for this part of the experiment. The earnings from this randomly-chosen round will be converted into Euro at the end of the experiment and paid out to you in cash. There are no participants present in the lab who take on the role of patients. An actual patient will benefit from the patient utility resulting from the quality of care you selected in the randomly-chosen round: A monetary value equalling the patient utility derived from your decision, multiplied by the number of patients who seek treatment from you, will be transferred to Christoffel Blindenmission Deutschland e.V., 64625 Bensheim. This organisation will use the funds to enable the treatment of patients suffering from cataracts, a serious eye condition.

Control questions. Before proceeding to the decisions in the experiment, we would like to ask you to answer several control questions. These control questions should make it easier

for you to become acquainted with the decision-making situation. If you have questions about this, please raise your hand. The first part of the experiment will begin after all participants have correctly answered the control questions.

Payment Procedure. In order to ensure that payments to the participants and the transfer of the monetary donation to Christoffel Blindenmission Deutschland e.V. are carried out correctly, an overseer will be randomly chosen after the third part of the experiment. The overseer receives a fee of EUR 5 in addition to his or her regular payment from the experiment. The overseer will affirm that the transfer to Christoffel Blindenmission is correctly carried out by the financial administration of the University of Cologne. For the transfer to Christoffel Blindenmission, the overseer will fill out a payment order to Christoffel Blindenmission with the amount, in Euro, that corresponds to the patient utility realized in the randomly selected round. The financial administration of the University of Cologne will then execute payment of the donation to Christoffel Blindenmission using funds allocated for this experiment. The form will be placed in a stamped envelope addressed to the financial administration of the University of Cologne. The overseer and the experimenter will jointly deposit this envelope in the nearest mailbox.

The overseer will confirm by signing a form that he or she properly carried out the assigned tasks, as described above. A copy of this form, as well as a copy of the confirmation from Christoffel Blindenmission that the donation was received, can be requested by all participants from the office of the Seminar of Personnel Economics and Human Resource Management. The copies will be sent by e-mail.
